# Data-driven platform for identifying variants of interest in COVID-19 virus

**DOI:** 10.1016/j.csbj.2022.06.005

**Published:** 2022-06-03

**Authors:** Priya Ramarao-Milne, Yatish Jain, Letitia M.F. Sng, Brendan Hosking, Carol Lee, Arash Bayat, Michael Kuiper, Laurence O.W. Wilson, Natalie A. Twine, Denis C. Bauer

**Affiliations:** a Australian e-Health Research Centre, Commonwealth Scientific and Industrial Research Organisation, New South Wales, Sydney, Australia; b Department of Biomedical Sciences, Macquarie University, New South Wales, Sydney, Australia; c Data 61, Commonwealth Scientific and Industrial Research Organisation, Canberra, ACT, Australia; d Garvan Institute of Medical Research, New South Wales, Sydney, Australia; e Applied BioSciences, Faculty of Science and Engineering, Macquarie University, New South Wales, Sydney, Australia

**Keywords:** GWAS, COVID-19, SARS-CoV-2, Case-control, Nsp14, AlphaFold

## Abstract

New SARS-CoV-2 variants emerge as part of the virus’ adaptation to the human host. The Health Organizations are monitoring newly emerging variants with suspected impact on disease or vaccination efficacy as Variants Being Monitored (VBM), like Delta and Omicron. Genetic changes (SNVs) compared to the Wuhan variant characterize VBMs with current emphasis on the spike protein and lineage markers. However, monitoring VBMs in such a way might miss SNVs with functional effect on disease.

Here we introduce a lineage-agnostic genome-wide approach to identify SNVs associated with disease. We curated a case-control dataset of 10,520 samples and identified 117 SNVs significantly associated with adverse patient outcome. While 40% (47) SNV are already monitored and 36% (43) are in the spike protein, we also identified 70 new SNVs that are associated with disease outcome. 31 of these are disease-worsening and predominantly located in the 3′-5′ exonuclease (NSP14) with structural modelling revealing a concise cluster in the Zn binding domain that has known host-immune modulating function. Furthermore, we generate clade-independent VBM groupings by identifying interacting SNVs (epistasis). We find 37 sets of higher-order epistatic interactions joining 5 genomic regions (nsp3, nsp14, Spike S1, ORF3a, N). Structural modelling of these regions provides insights into potential mechanistic pathways of increased virulence as well as orthogonal methods of validation.

Clade-independent monitoring of functionally interacting (epistasis, co-evolution) SNVs detected emerging VBM a week before they were flagged by Health Organizations and in conjunction with structural modelling provides faster, mechanistic insight into emerging strains to guide public health interventions.

## Highlights

• We provide an automated way to identify emerging variants of concern using viral genome and patient-outcome data.
• We assembled 10,000 sample-strong case-control dataset and identified 117 single nucleotide variants (SNV) associated with adverse patient outcomes.
• We observe co-evolution of protective and pathogenic interactions between the spike, nsp14, and N region with either orf3a or nsp3.
• Structural modelling reveals mutation clusters in the Zn binding domain of nsp14 suggesting ongoing adaptation to the human host.
• Our approach identified Variants Being Monitored (VBM) a week before they were flagged by Health Organizations and offers a clade-independent function-orientated grouping.

## Graphical abstract

**Figure:**
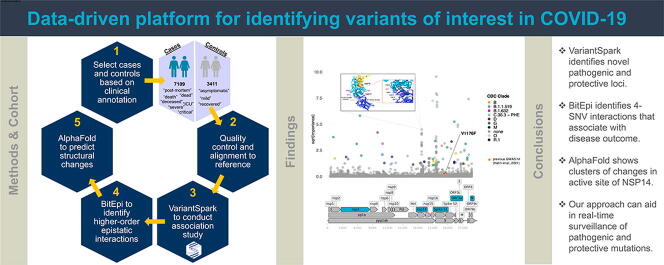

## 1 Introduction

Genetic mutations of SARS-CoV-2 have emerged as part of the virus’ adaptation to the human host. There is evidence that some of these mutations have made the virus more transmissible, have caused more severe disease, or reduced diagnostics, treatments, or vaccine effectiveness. Virus strains containing mutations with functional consequences are catalogued by the Centers for Disease Control and Prevention (CDC) as Variants of Concern (VOC) [1]. Examples include the Delta Variant, which is characterized by 15 single nucleotide variants (SNVs) in the spike protein and the Omicron Variant, characterized by 37 SNV in the spike protein.

CDC also defined ‘Variants of Interest’ (VOI), for which there is emerging evidence that implies their role in changed receptor binding, reduced neutralization by antibodies generated against previous infection or vaccination, reduced efficacy of treatments, potential diagnostic impact, or predicted increase in transmissibility or disease severity. To monitor potential VOI and/or VOC, CDC maintains a list of ‘Variants Being Monitored’ (VBM) with their specific phylogenetic lineages with examples being Alpha, Beta, Gamma, Epsilon, Eta, Iota, Kappa, Zeta, Mu. All VBM to date have focused on the genomic regions of the spike protein as it is the most well understood segment of the virus and where *in vivo* and protein structure experiments can best provide evidence of functional changes [2].

However, there is evidence that other regions of the SARS-CoV-2 virus may also have an impact on clinically relevant properties [3], [4]. A genome-wide screening for SNVs with genome-phenome association, such as severity of disease, is hence desirable to gain the full picture of existing and emerging VBM.

Traditional genome-wide association studies (GWAS) can identify SNVs that are statistically associated with common or complex traits using regression-based approaches [5]. Indeed, a GWAS study on 7,548 patient-outcome annotated SARS-CoV-2 samples from the Global Initiative on Sharing All Influenza Data (GISAID) used logistic regression to identify SNVs associated with disease outcome. Surprisingly, they identified only a single locus of significance (25088 bp resulting in V1176F in the Spike protein) [6]. Since VBMs are characterized by multiple SNVs it is hence more likely that multiple loci in the viral genome evolve together to modulate its pathology and such an outcome would have been expected in a GWAS study.

The reason for this unexpected outcome might be that these genomic changes individually only have small or no functional effects, and only when taken together explain the different capabilities of the viral strains (epistasis). Traditional methods, like logistic regression, are hence not suitable to identify such epistatic interactions of SNV with small effect size.

Here, we introduce VariantSpark as a platform for the automatic detection of genome-wide interacting SNV in large international data resources with the ability to characterize emerging VBM. VariantSpark, originally developed for the human disease space [7], is a distributed machine learning framework capable of identifying complex genomic associations and was adapted to identify SNV with likely functional consequences in SARS-CoV-2 genomes. Unlike other Random Forest packages such as Ranger [8], VariantSpark can process very large datasets and can handle ambiguity codes needed to process non-human encodings.

Demonstrating the power of this approach, we assembled the largest association dataset to date with 10,000 case-control samples by carefully curating the “Patient Status” field from GISAID and matching it with the mutation profile of the viral genome. We used VariantSpark on this stringent case/control dataset to identify SNVs associated with severe disease outcome. We annotate the set of SNVs which are jointly driving disease using BitEpi [9], a software for the exhaustive search of up to 4 epistatic interaction partners, to generate clade-independent definitions of VBMs.

## 2 Results and discussion

### 2.1 Curating the largest Case-Control dataset for VOI detection

We first assembled the case-control dataset by obtaining the “Patient Status” field of the 3,472,078 GISAID samples (data freeze on 14th Sep 2021). To curate a high-confidence dataset, we group the samples by patient outcome into 3411 cases (worse disease outcome) and 7109 controls. Table 1 summarises our inclusion and exclusion criterion. Note that only 0.3% of samples passed our inclusion criteria despite GISAID making the “Patient Status” field mandatory on 27 April 2020, indicating an ongoing issue with data standardization [10]. During the quality control step, we removed a further 276 samples, which had incomplete genomic information (sequence length less than 29000) or sequences from non-human sources (e.g. pangolin). To our knowledge, this resulted in the world's largest case-control dataset for VBM detection with 10,520 samples.

**Table 1:** Summary of sample inclusion into the case-control dataset.

*Annotations*	*Removed/Kept*	*Number of Samples*
Patient status annotated as ‘Unknown’	Removed	3,312,914
Ambiguous annotations that cannot be associated with better or worse disease outcome including, ‘Live’, ‘Hospitalized’, ‘Outpatient’, ‘Symptomatic’, ‘Released’, ‘Ambulatory’, ‘Inpatient’, ‘other’.	Removed	120,429
Unannotated (missing patient status)	Removed	27,939
‘Deceased’, ‘Severe’, ‘Critical’, ‘Dead’, ‘Post-mortem’, ‘Death’ and ‘ICU’.	Kept – Cases	3,639
‘Asymptomatic’, ‘Mild’, ‘Mild clinical signs without hospitalisation’, and ‘Recovered’	Kept – Controls	7,157
Total Samples Removed		**3,461,282**
Total Samples Kept		**10,796**

### 2.2 Estimating the effects of confounders

Next, we evaluated if this new case-control dataset had any geographical biases. For example, whether samples from regions with relatively poor healthcare may be overrepresented in the cases while countries with higher sequencing and reporting regimes may skew the control samples.

While a large proportion of reporting countries had an even distribution of cases and controls, some countries, like Bulgaria were indeed overrepresented in the cases, while Réunion island was enriched in controls. This suggests that geographical bias is not the main driver for clustering.

To test this hypothesis, we compared the data clustering with country against clustering with the dominant variant or clade which was circulating at the time. We first conducted principal component analysis to reduce the dimensionality of the data, resulting in 29 principal components accounting for 99.9% of the total variance explained. Next, we performed Uniform Manifold Approximation and Projection (UMAP) [11] to visualise our data. We then used density-based spatial clustering of applications with noise (DBSCAN) to cluster our data, resulting in 8 distinct clusters **(**Supplementary Figure S1**)**. Color-coding revealed an association with CDC clade **(**Supplementary Figure S2**)** instead of country **(**Supplementary Figure S3**)**. To quantify this, we calculated the purity and entropy.

The purity and entropy of the clade clustering were 0.698 and 0.446 respectively, where 1 represents a strong relationship between the clustering and the annotation. In contrast, clustering by country only achieved a purity of 0.429 and entropy of 0.291. Similarly, the adjusted-rand index also suggested a stronger relationship with clades (0.247) rather than country (0.104). Taken together, these data suggests that the overrepresentation of cases/controls in some regions are a consequence of the genetic make-up of the virus strains active in the region rather than a data collection artefact.

### 2.3 VariantSpark identifies novel single nucleotide variants associated with patient outcome

We conducted the case-control study on the 10,520 samples to identify genetic variants that are associated with poor health outcome (see Table 1). We used VariantSpark to determine the Gini-importance score for all genetic variants in the viral genome. In order to maximise the accuracy of our model, parameter tuning was conducted to determine the optimum parameters for our analysis. Out of bag (OOB) error was used to estimate model accuracy, resulting in a minimum OOB of 0.251 based on the parameters tested **(**Supplementary Table S1**)**. All further analyses were carried out using the selected parameters **(**Supplementary Table S1**, green highlight)**. We performed significance testing by measuring the deviation of the observed Gini importance scores from the right-skewed distribution of the background signal [12].

**Fig. 1:**
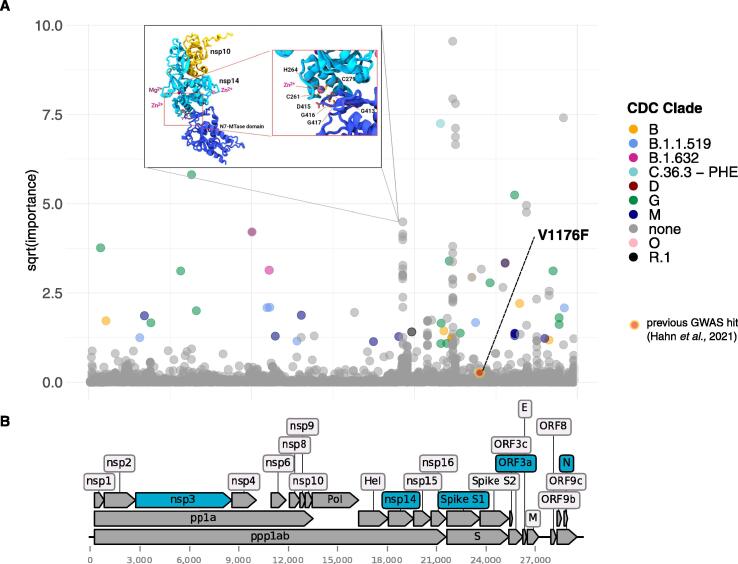
**Results from association analysis. A)** Manhattan plot of VariantSpark gini importance scores with 10,520 case/control data. 100 bp are removed on each end. Mutations associated with current and previous variants being monitored (VBM) are labelled and coloured while mutations which are not currently associated with a VBM are grey and unlabelled. Red dot with yellow border represents hit from a previous GWAS study (Hahn et al., 2021). **B)** SARS-CoV-2 genome and regions corresponding to protein regions. Protein regions coloured in dark red correspond to protein regions with significant clusters of mutations (from Fig. 1A). Protein regions highlighted in blue represent regions involved in putative highly associative 4-SNV interactions. Inset represents AlphaFold prediction and location of amino acid residues corresponding to the nsp14 mutation cluster identified by VariantSpark. (For interpretation of the references to color in this figure legend, the reader is referred to the web version of this article.)

We identified 117 genetic mutations that had a significant association with patient health outcome (FDR adjusted p-value cut off 0.01, Supplementary Table S2). As shown in Fig. 1, of the 117 significant SNV 36% (43/117) are located in the spike protein and 40% (47/117) are already monitored in one or more VBMs (16 mutations are reported in the Gamma variant, 12 in the Mu, 11 Beta, 1 in the Delta and 9 in other VBM).

To investigate the relevance of the 70 mutations not currently monitored as VBMs, we next identified their likely role on disease outcome, e.g., protective (mild disease) or pathogenic (severe disease). We calculated the odds ratio with 95% confidence interval to classify loci as protective (confidence interval below 1) or pathogenic (confidence interval above 1). We identified 64 SNV to be pathogenic and 53 SNV to be protective amongst the 117 significant SNVs. Unsurprisingly, 70% of the SNVs included in the VBM are pathogenic (33/47). However, there are also 14 protective SNVs defining the VBMs. Conversely, out of the 70 SNV that are not currently part of VBM, 31 have putative pathogenic effects **(**Table 2**)**.

**Table 2:** VariantSpark predicted 31 novel variants associated with worse disease outcome.

Locus	REF	ALT	p-value	Gene	Consequence	Product
19,276	G	N	4.75E-07	NSP14	'G413S', 'G413R', 'G413C'	3′-to-5′ exonuclease
19,277	G	N	9.08E + 00	NSP14	'G413D', 'G413A', 'G413V'	3′-to-5′ exonuclease
19,278	T	N	9.14E + 00	NSP14	'G413G', 'G413G', 'G413G'	3′-to-5′ exonuclease
19,279	T	N	9.03E + 00	NSP14	'C414S', 'C414R', 'C414G'	3′-to-5′ exonuclease
19,280	G	N	2.34E-06	NSP14	'C414Y', 'C414S', 'C414F'	3′-to-5′ exonuclease
19,281	T	N	2.10E-06	NSP14	'C414*', 'C414C', 'C414W'	3′-to-5′ exonuclease
19,282	G	N	1.54E-06	NSP14	'D415N', 'D415H', 'D415Y'	3′-to-5′ exonuclease
19,283	A	N	4.36E-07	NSP14	'D415A', 'D415G', 'D415V'	3′-to-5′ exonuclease
19,284	T	N	5.29E-07	NSP14	'D415E', 'D415D', 'D415E'	3′-to-5′ exonuclease
19,285	G	N	2.98E-07	NSP14	'G416S', 'G416R', 'G416C'	3′-to-5′ exonuclease
19,286	G	N	1.34E-06	NSP14	'G416D', 'G416A', 'G416V'	3′-to-5′ exonuclease
19,287	T	N	5.62E-06	NSP14	'G416G', 'G416G', 'G416G'	3′-to-5′ exonuclease
19,288	G	N	9.62E-06	NSP14	'G417S', 'G417R', 'G417C'	3′-to-5′ exonuclease
20,800	A	N	6.14E-05	NSP16	'T48P', 'T48A', 'T48S'	2′-O-ribose methyltransferase
20,801	C	N	5.22E-05	NSP16	'T48N', 'T48S', 'T48I'	2′-O-ribose methyltransferase
20,802	T	N	6.71E-05	NSP16	'T48T', 'T48T', 'T48T'	2′-O-ribose methyltransferase
20,803	C	N	6.68E-05	NSP16	'Q49K', 'Q49E', 'Q49*'	2′-O-ribose methyltransferase
20,804	A	N	6.85E-05	NSP16	'Q49P', 'Q49R', 'Q49L'	2′-O-ribose methyltransferase
20,805	A	N	6.46E-05	NSP16	'Q49H', 'Q49Q', 'Q49H'	2′-O-ribose methyltransferase
20,809	T	N	2.21E-05	NSP16	'C51S', 'C51R', 'C51G'	2′-O-ribose methyltransferase
20,810	G	N	2.16E-05	NSP16	'C51Y', 'C51S', 'C51F'	2′-O-ribose methyltransferase
20,811	T	N	2.25E-05	NSP16	'C51*', 'C51C', 'C51W'	2′-O-ribose methyltransferase
20,812	C	N	2.31E-05	NSP16	'Q52K', 'Q52E', 'Q52*'	2′-O-ribose methyltransferase
20,813	A	N	2.18E-05	NSP16	'Q52P', 'Q52R', 'Q52L'	2′-O-ribose methyltransferase
26,492	A	T	5.93E-05	Between E and M region		
27,512	A	N	1.03E-04	ORF7a	'Y40S', 'Y40C', 'Y40F'	Accessory protein
27,513	C	N	1.02E-04	ORF7a	'Y40*', 'Y40*', 'Y40Y'	Accessory protein
27,514	G	N	1.06E-04	ORF7a	'E41K', 'E41Q', 'E41*'	Accessory protein
27,516	G	N	7.21E-05	ORF7a	'E41E', 'E41D', 'E41D'	Accessory protein
28,272	A	T	4.13E-06	Between ORF8 and N region		
29,782	A	*	8.62E-05	N/A		

With the global health organizations focusing on the spike protein, it is noteworthy that all of the 31 unmonitored and putatively pathogenic SNVs occur in other regions of the genome, namely ORF1ab (which produces either 3′ to 5′ exonuclease nsp14 or 2′ O-ribose methyl transferase nsp16) and ORF7a (interferon antagonist). nsp14 and nsp16 along with the stimulatory factor nsp10 is important for viral replication, RNA stability and RNA viral proofreading [13], [14]. Interestingly, SARS-CoV-2 ORF7a ectodomain has been found to bind efficiently to human CD14^+^ monocytes, suggesting that SNVs in this region may differentially modulate the severity of the host immune response to viral infection [15]. Monocytes are a key driver in the recruitment of macrophages to the lungs, and increased levels of macrophages have been shown to correlate with increased disease severity [15]. Taken together, this suggests that SNV outside the spike protein need to be monitored.

### 2.4 VariantSpark hits are robust and replicable with logistic regression.

To technically validate our findings, we compared the VariantSpark hits with results from Firth’s logistic regression including the first 20 principal components as covariates. We next conducted Spearman’s rank correlation to compare the ranks from VariantSpark hits and hits from logistic regression. The rank correlation for the top 20 LR hits was 0.90, indicating a good agreement on the dominant signal. As expected, the rank correlation for the top 100 hits reduced to 0.68 because LR is not able to take gene-gene interactions into account. Drilling in further, we found that the two main clusters of hits we identified in nsp14 and spike regions with VariantSpark overlapped with the LR clusters **(**Supplementary Figure S4**,** Supplementary Tables S4 **–** S6**)**.

We further tested the robustness of the results by creating a down-sampled balanced dataset (1:1, case:control), as random forest methods like VariantSpark are sensitive to imbalanced training data. We ran VariantSpark on a dataset with 3412 cases and 3714 controls. Supplementary Table S3 summarizes the comparable number of top 100 hits in both balanced and actual dataset. We determined that clusters of mutations in S proteins were reproduced. This finding suggests that for this analysis, the impact of our original imbalanced dataset is likely minimal. Therefore, we retained the largest dataset available to avoid any data loss due to under-sampling.

### 2.5 Disease associated SNV have epistatic interactions and structural changes

Next, we investigated which of the 117 SNV have epistatic interaction and jointly modulate disease outcome. Using BitEpi we identified 99 highly associative 2-SNV interactions with all the hits comprising of 1 protective SNV and 1 pathogenic SNV indicating a balanced co-evolution **(**Supplementary Table S7**)**.

**Fig. 2:**
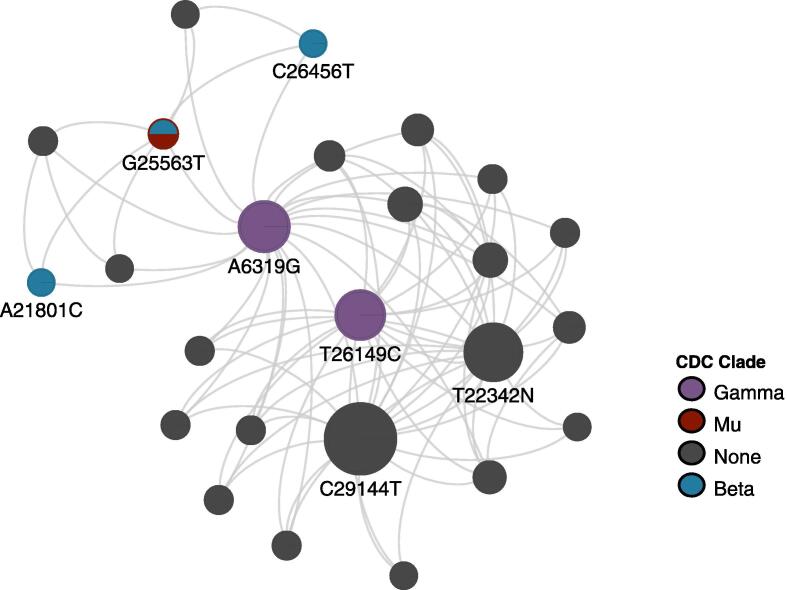
**Network of 4-SNV combinations showing highly associative interactions.** Coloured nodes indicate SNVs found in VBM. Size of node is proportional to the frequency at which that SNV is involved in highly associated 4-SNV interactions.

To investigate this behaviour further, we looked at higher-order interactions. BitEpi identified 540 highly associative 3-SNV interactions **(**Supplementary Table S8**)** and 37 4-SNV interactions **(**Fig. 2**,** Supplementary Table S9**)**, respectively. 92% (34/37) of the 37 4-SNV interactions involved interactions between nsp14 region, spike region, N region with either ORF3a region or nsp3 region. To investigate the co-evolution property we constructed contingency tables for the 4-SNVs, which lists the number of cases versus controls of each of the involved genotypes. From this we can identify which particular genotypes in the 4-SNV interactions are more frequently observed in cases versus controls by determining the deviation from the over-all case-ratio, which is 0.37. Each SNV case-rate is listed in Supplementary Figures S5 – S7**,** Supplementary Table S10.

We again find interactive pairs of protective/pathogenic co-evolution. For example, the 4-SNV combination of 6319A:21801A:22346G:25563 T with one alternative allele “0001” seems to be very pathogenic, doubling the case-ratio over the baseline (0.63 vs 0.37) **(**Supplementary Table S11**)**. However, pairing this with another alternative allele “0011”, with a shift away from G at 22346, reduces the pathogenic effect to 0.45, because this mutation alone seems to be very protective (“0010” has a case ratio of only 0.08).

We examined the distribution of these allele combinations across viral strains/clades in a recent data freeze from GISAID (22nd March 2022). Interestingly, we found that although some allele combinations were quite specific to a particular CDC variant, most allele combinations were distributed over multiple variants. For example, of all the samples with the pathogenic D_1 (**6319A:21801A:22346 N:25563 T**) combination, 98.9% were comprised of the Beta variant **(**Supplementary Table S12**)**. More commonly, allele combinations were not variant-specific, with 73.8% of all samples with the pathogenic B_1 combination (**6319A:21801A:22346G:25563 T)** classified into the mixed group “Other” and the protective C_1 combination (**6319A:21801A:22346 N:25563G**) evenly distributed between Alpha, Omicron, Delta and Other groups. This effect was observed in most of the significant pathogenic and protective combinations we investigated **(**Supplementary Table S13**)**. This co-evolution of protective and pathogenic SNV further substantiates that variants should be monitored independent of their phylogenetic clade membership and rather based on their functional association with phenotype and other SNV. Further *in-vitro* and *in-vivo* studies are needed to establish the functional importance of interactions between these regions.

### 2.6 Predicted structural consequences of pathogenic mutations

In this section we investigate the potential consequences of the identified VariantSpark mutations. We focused on the 31 unmonitored pathogenic mutations to focus the discussion.

Interestingly, 29 of the 31 unmonitored pathogenic mutations, and indeed 63 out of the 117 significant VariantSpark hits, resulted in an allele change to “N”. This indicates that any move away from the original Wuhan strain has an influence on the disease outcome. An observation that is consistent with a virus under substantial selective pressure and evolutionary activity after the jump to a new host. To investigate this further we use NextVariant, a script to list the codon changes that are associated with such changes.

Table 2 lists the predicted consequences for any associated proteins. These were predominantly amino-acid substitutions, but we also found 1 deletion and two SNVs in intergenic regions. We found that the mutations clustering around the 3′-5′ exonuclease had the highest importance scores of all the significant pathogenic loci. Interestingly, these mutations cluster around codons 413–415, which represent the active site of the 3′-5′ exonuclease, containing a metal binding domain. Again, this is consistent with evolutionary pressure on a non-optimal active site for human hosts. We also found 3 silent mutations. Previous studies have highlighted the presence of synonymous mutations in nsp16 that show a high rate of positive selection, suggesting that although such mutations may not change the amino acid sequence, changes to the RNA secondary structure may affect other cellular functions [16], [17].

**Fig. 3:**
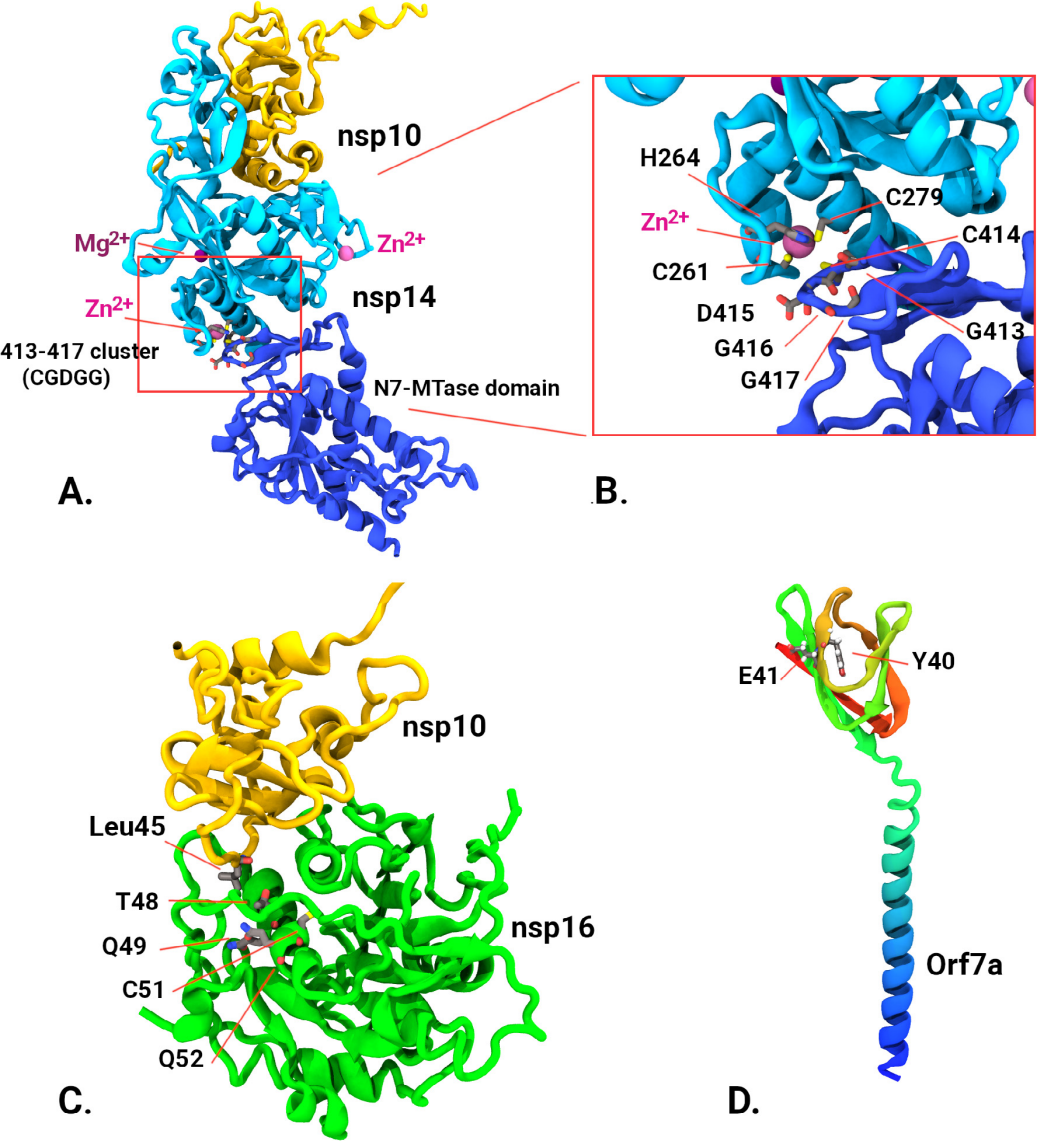
**Structural analysis of protein models.** Alphafold models (verified with crystallographic data where possible). **A)** 3′-5′ exonuclease (nsp14) (cyan and blue) complexed with nsp10 (yellow) showing relative positions of 413:417 cluster in the N7-MTase domain to Zn binding residues and other ion binding sites. **B)** Close up of the 413–417 cluster in nsp14 showing proximity of Zn binding domain. **C)** Structure of nsp10/nsp16 complex (from pdb; 6W4H and Alphafold models) showing nsp16 mutational cluster (T48, Q49, C51, Q52) and its proximity to nsp10 binding, in particular with residue Leu45 form nsp10. **D)** Predicted Alphafold model of Orf7a accessory protein showing putative mutation sites Y40 and E41. (For interpretation of the references to color in this figure legend, the reader is referred to the web version of this article.)

To further assess structural implications of these mutational changes we evaluated models of both crystallographic data augmented with AlphaFold2 structure predictions [18] **(**Fig. 3**)**. As both the 3′ to 5′ exonuclease (nsp14) and 2′-O-ribose methyltransferase (nsp16) are found as independent allosteric complexes with nsp10 we modelled the respective heterodimers. Alphafold2 model predictions agreed remarkably well with crystallographic data with RMSD differences of less than 0.95 Å to where crystallographic data existed but had the advantage of including regions missing in the diffraction data. Our modelling showed that the observed cluster of mutations in nsp14 including 413 to 417 (residue sequence GCDGG) occurs in the S-adenosyl methionine (SAM)-dependent (guanine-N7) methyl transferase domain (N7-MTase) at the junction on the N-terminal domain and is adjacent to the zinc binding motif from residues His 257, Cys261, His264 and Cys279. In the case of nsp16, the cluster of mutations T48, Q49, C51, Q52 occurred at the interface of nsp10, with T48 being adjacent to leucine 45 of nsp10 potentially altering the strength of the nsp10/16 complex formation and subsequent activity kinetics. Orf7a accessory protein contains two putative adverse outcome associated mutation at position Y40 and E41. This protein is thought to interfere with a human defence protein tetherin, by glycosylation interference which is thought to enhance viral escape and proliferation [19]. As no Orf7a/tetherin structure currently exists we were not able to investigate this interaction further.

The structural analysis suggests that the locations of the observed putative mutation sites could plausibly modulate the activity of key viral proteins of nsp14, nsp16, and their interacting partner nsp10. Though our sequence analysis does not provide specific mutations, changes in the highlighted positions would be expected to change the strength of protein–protein interactions in the case of nsp10/14, or even alter the flexibility of inter-domain interactions as in the case of nsp16. We are unable to determine the effects that this may have on viral-host interactions in the scope of this study.

## 3 Conclusion

New SARS-CoV-2 variants continue to emerge giving rise to the need for a data-driven platform that can flag SNV with functional consequences early. Our combination of machine learning and structural modelling may offer such a solution. Specifically, CDC started monitoring the Mu variant on Sep 21, 2021 and VariantSpark flagged 12 mutations characterizing Mu working with Sep 14th data. Similarly, VariantSpark flagged C27513T SNV, which WHO started monitoring as B.1.640 originating in Republic of Congo.

Moreover, VariantSpark identified new SNV with statistically significant association on disease outcome. It might therefore be more informative to use these SNVs for tracking and differentiating VBMs rather than lineage markers, which might not have functional consequences. Sets of disease associated SNV, especially when they are shown to interact with each other through a BitEpi analysis, may provide insights into the molecular cause for the different capabilities and pathology of VBMs.

Our work shows protein model analysis, such as those provided by crystallography and AlphaFold, can be a useful addition to sequence analysis by providing structural context of mutations, both within the protein and its binding partners. These insights can help determine if the observations are plausible and may help mechanistic interpretations.

For example, recent laboratory experiments have highlighted protein residues crucial to the translation inhibition activity of NSP14′s exonuclease domain, such as C261 [20], [21], [22]. Using AlphaFold we can see that the VariantSpark-identified mutations, Cys 414 and Asp 415, (Supplementary Table S5) are adjacent to laboratory-evaluated C261 (Fig. 3**, inset**). It is likely that the mutations observed in epidemiological data have modulating effects on the zinc finger motif and thus the translational inhibition capability of SARS-CoV-2.

Using genomic, health, structural and molecular data, our study provides further evidence supporting the importance of this region as an attractive therapeutic target for SARS-CoV-2 [23] e.g. by inhibiting this complex, which increases efficacy of antiviral drugs such as remdesivir [24]. It also provides evidence for the ongoing evolutionary activity of the virus in adapting to its new human host. We noted the disease associated SNVs around the active site of 3′-5′ exonuclease and the observation that any shift away from the original Wuhan-allele had impact on disease outcome.

While this is the largest case-control dataset assembled to date, it is far from being sufficient for the robust automatic surveillance of emerging VBM. More than 99% of GISAID samples were lost due to the lack of annotations for patient outcome. This means that our results may be impacted by sampling bias, as the samples included in our study may not necessarily be representative of the whole dataset. This emphasises the crucial need for improved clinical annotations in databases such as GISAID. Ideally, location-matched samples should be used to avoid reporting bias. For example, we noticed collection sites, which submitted more samples from deceased patients than asymptomatic patients. While this seem to have been averaged out in our global analysis (see Section 3.2), a local or country-specific analysis would not be possible. Another caveat of our study is that we have used a single variable, the viral genome, to predict disease outcome. In reality, patient outcome would be the result of a complex interplay between viral strain, hospital care and patient characteristics such as age, immune system and comorbidities.

Despite the shortcoming of a limited dataset, it is encouraging that our analysis identified mutations associated with known variants of concern, including the 25088 bp locus identified in previous studies [6] as well as suggested novel mutations for monitoring. With additional analysis of crystallographic structures augmented with Alphafold models of protein complexes, we could predict the importance of lesser-known mutations based on their structural context, e.g. for NSP14. Our method of identifying single mutations and 2-, 3- and 4-SNV combinations that significantly affect patient outcome and are supported by protein modelling predictions may offer a streamlined approach to quickly flag dangerous mutation combinations and has the potential to supplement current variant surveillance efforts. Future work should include *in vitro* assays assessing functional consequences of the novel mutations identified in this study.

## 4 Methods

New SARS-CoV-2 sequences are added constantly to GISAID’s central repository of SARS-CoV-2 genomes. We took a data freeze on 14th Sep 2021 to work with 3,444,139 sequences. 3,306,730 of these sequences had “unknown” annotation for patient status field.

### 4.1 Data wrangling

We started curating the remaining 1,37,409 sequences to identify datasets with severe disease outcomes and no/less disease outcomes for cases and controls respectively. For cases, we used the patient status of “deceased”, “severe”, “critical”, “dead”, “post-mortem”, “death” and “ICU” with a total of 3639 sequences. For control, we used the patient status of “Asymptomatic”, “mild”, “Mild clinical signs without hospitalization” and “recovered” with a total of 7157 sequences. We then ran the 10,796 sequences to quality control (QC) process and removed the incomplete sequences (sequence length not 29000) and sequences not from human source. After QC the final dataset comprised of 3411 cases and 7109 controls with a total of 10,520 sequences with appropriate patient status annotations.

### 4.2 Data reformatting

VariantSpark accepts the locus information in VCF format and a corresponding label file associating VCF file sample names to phenotypes. To generate the VCF file for 4161 sequences we first aligned these to WIV04 reference sequence using MAFFT [25] (v7.471) alignment. The alignment was then converted to VCF format using the snp-sites (v2.3.3) [26] and the perl script was used to reset the reference used by MAFFT. The vcf file compressed by bgzip was used as input to VariantSpark. Sample names were isolated from the vcf file and were tagged against cases and controls as 1 and 0 respectively.

### 4.3 Exploratory data analysis

Principal component analysis was conducted using the R package *PCAtools* [27]. We retained all principal components accounting for 99.9% of the total variance for our dataset. Following this, the R package *umap* [28] was used to perform Uniform Manifold Approximation and Projection on the principal components derived from the previous step. External cluster validation and DBSCAN clustering was conducted using the R package *fpc* [29] using an optimal epsilon value of 0.45, and the threshold for minimum number of points per cluster was set at 100. The python package DNA Features Viewer [30] was used to produce images with SARS-CoV-2 protein regions.

### 4.4 VariantSpark and logistic regression association analyses

VariantSpark analysis was conducted on an AWS EMR through the RONIN interface with the following configurations: a Ubuntu 18.04 LTS server to run a BioSpark cluster on 8 × c5n.large instance with 5.25 Gb of RAM and 2vCPUs (total of 42 Gb of RAM and 16vCPUs). Hail 2.0 was used to construct matrix tables from our VCF file and phenotype data for use in VariantSpark. The multi-allelic VCF was split using *bcftools* [31] (version 1.12) to allow for allele-specific associations*. p*-values for VariantSpark analysis were computed using the R package RLocalFDR, which uses a Bayesian approach to calculate thresholds for gini importance scores (https://doi.org/10.1101/2022.04.06.487300). Firth’s logistic regression was conducted in Hail 2.0, using the first 20 principal components as covariates. Spearman’s rank correlation was used to compare ranked hits between VariantSpark and logistic regression methods.

### 4.5 Odds ratio analysis

The odds ratio of each locus’ association with cases was calculated, including a 95% confidence interval.. The odds ratio represents the relative likelihood of a sample being a case, given that it has a variant at a given locus. It is calculated as shown below, where *c_1_* and *c_0_* are the numbers of cases with and without the variant respectively, and *n_1_* and *n_0_* are the numbers of controls with and without the variant respectively.

(1) $$\mathrm{oddsratio}=\frac{c_{1}\cdot n_{0}}{c_{0}\cdot n_{1}}$$

To give a confidence interval of 95% we then calculated the upper and lower bound for this odds ratio using the following formulae, where *OR* is the odds ratio as calculated above.

(2) $$upper95\%CI=OR\cdot e^{1.96\cdot \sqrt{\frac{1}{c_{0}}+\frac{1}{c_{1}}+\frac{1}{n_{0}}+\frac{1}{n_{1}}}}$$

(3) $$lower95\%CI=\frac{\mathrm{OR}}{e^{1.96\cdot \sqrt{\frac{1}{c_{0}}+\frac{1}{c_{1}}+\frac{1}{n_{0}}+\frac{1}{n_{1}}}}}$$

These calculations were repeated for each locus, and were used to classify a locus as protective, if the upper bound on the confidence interval was below 1, or pathogenic, if the lower bound was above 1. If the 95% confidence interval for a locus encompassed 1, the locus was discarded as not being insignificant.

### 4.6 NextVariant analysis

NextVariants script uses NCBI reference sequence NC_045512.2 as reference for SARS-CoV-2 genome. Using BioPython the script then maps any variants with nucleotide positions to reference sequence. Script then further identifies the corresponding gene, consequence, and product from the CDS section of genbank file. Consequences for “N” nucleotide is calculated based on codon changes by replacing N with A,T,G,C.

### 4.7 BitEpi analysis

More detailed information regarding the functionality and methodology of BitEpi has been described by Bayat *et al.* [9]. Briefly, BitEpi was used to identify 2-SNV, 3-SNV, and 4-SNV interactions associated with worse disease outcomes between the 117 significant VariantSpark SNVs. 228 highly associative 2-SNV, 1102 highly associative 3-SNV, and 43 highly associative 4-SNV were filtered based on thresholds of 95%, 99%, and 99.9% for 2- SNV, 3- SNV, and 4-SNV alpha and beta association effect respectively. Finally, after computing *p*-values for these filtered interactions, interactions with significant *p*-values after Bonferroni correction at 5% were kept. These interactions represent the final set of statistically significant highly associated interactions. Once we identify the 4-SNV combinations with significant beta and alpha values using BitEpi, we look further into those interactions by producing their corresponding contingency table. Using this table, one could identify the 'risk' and 'protective' allele combinations. While the 4-SNV contingency table can explain why beta value is significant. To understand why alpha value is significant we compare the best sub 3- SNV contingency table (highest 3-SNV beta) with the 4- SNV contingency table. If [A, B, C, D] is the 4- SNV interaction then (A, B, C), (A, B, D), (A, C, D) and (B, C, D) are possible sub 3- SNV combinations.

### 4.8 Structural modelling analysis

Crystallographic models of nsp14/nsp10 (pdb entry:7DIY [21]), nsp16/nsp10 (pdb entry: 6W4H [32]), and Orf7a (pdb entry: 6 W37 [33]) were visualized using VMD [34]. Additional model of the same sequences was generated using Alphafold2 [18]. Alignments of crystallographic data to AlphaFold models using VMD scripting showed good agreement with RMSD values of less than 0.95 Å. VMD was used for visual inspection of mutation sites and their proximity to protein interfaces and ion binding sites.

## Declaration of Competing Interest

The authors declare that they have no known competing financial interests or personal relationships that could have appeared to influence the work reported in this paper.
