# Supplementary material for: Data-driven platform for identifying variants of interest in COVID-19 virus
Source: Comput Struct Biotechnol J. 2022 Jun 3;20:2942–50. doi: 10.1016/j.csbj.2022.06.005 (PMC9162986; doi:10.1016/j.csbj.2022.06.005)

Supplementary Figure Legends

Supplementary Figure S1: UMAP visualisation of full dataset coloured by DBSCAN clusters. Points which are unassigned to a cluster (Cluster 0) are coloured in gray.

Supplementary Figure S2: UMAP visualisation of full dataset coloured by CDC clade.

Supplementary Figure S3: UMAP visualisation of full dataset coloured by country.

Supplementary Figure S4: Comparative Manhattan plots showing p_{-log10} values for logistic regression results and importance scores for VariantSpark results. LR; Firth's logistic regression, VS; VariantSpark

Supplementary Figure S5: Association metrics for 4-SNV combination 4-SNV-1 (6319:21801:22346:25563). 0 denotes reference allele, and 1 denotes the alternate allele for each 4-SNV locus. Rate refers to the ratio of controls:cases in that particular allele combination subset, whereas the purity refers to the purity of the control pool of samples in the subset. Set rate and set purity denote the ratio of controls:cases and purity of controls for the whole dataset.

Supplementary Figure S6: Association metrics for 4-SNV combination 4-SNV-2 (19288:22343:26149:29144). 0 denotes reference allele, and 1 denotes the alternate allele for each 4-SNV locus. Rate refers to the ratio of controls:cases in that particular allele combination subset, whereas the purity refers to the purity of the control pool of samples in the subset. Set rate and set purity denote the ratio of controls:cases and purity of controls for the whole dataset.

Supplementary Figure S7: Association metrics for 4-SNV combination 4-SNV-3 (6319:19288:22343:29144). 0 denotes reference allele, and 1 denotes the alternate allele for each 4-SNV locus. Rate refers to the ratio of controls:cases in that particular allele combination subset, whereas the purity refers to the purity of the control pool of samples in the subset. Set rate and set purity denote the ratio of controls:cases and purity of controls for the whole dataset.

Supplementary Figure S1

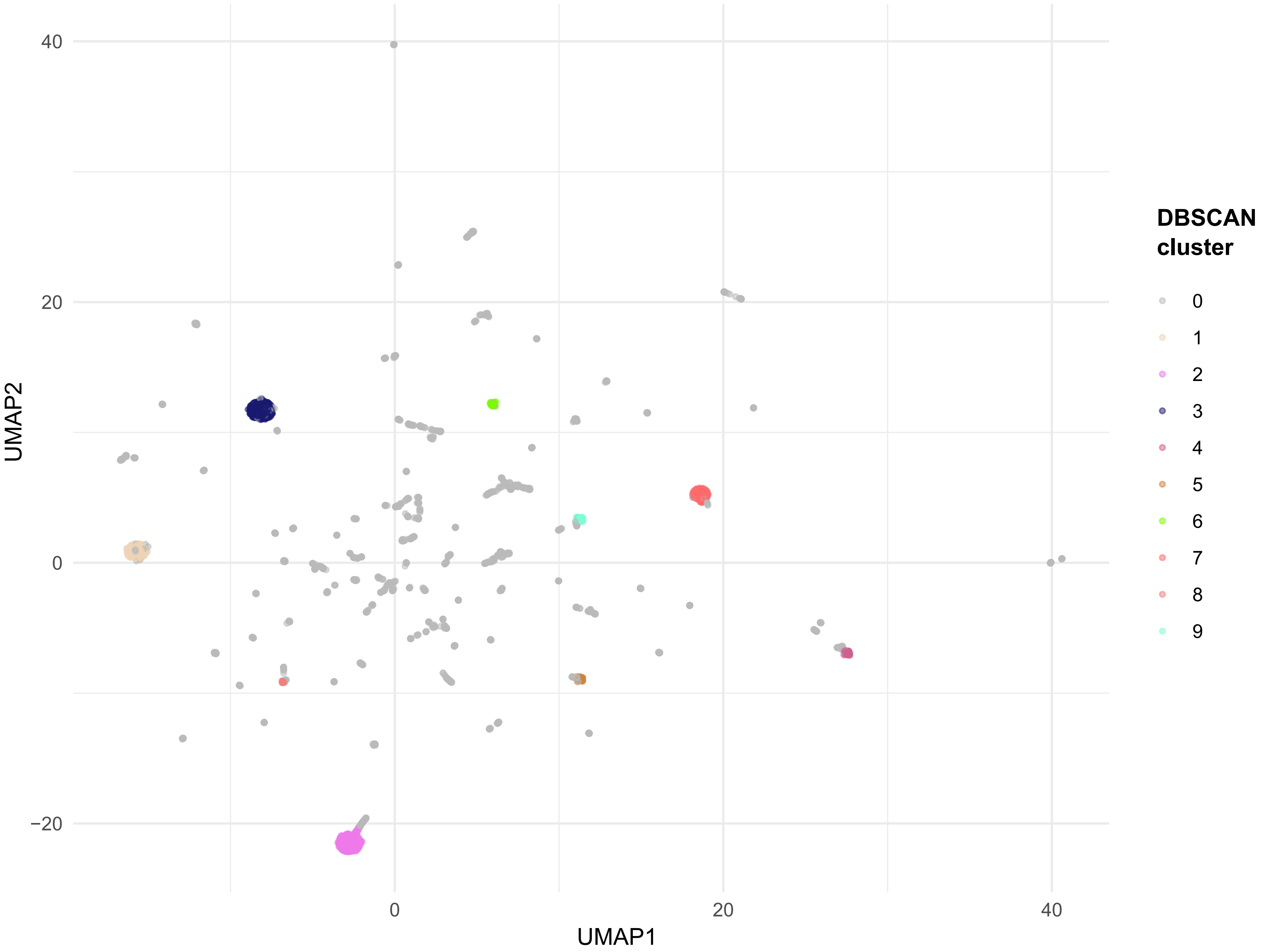

Supplementary Figure S2

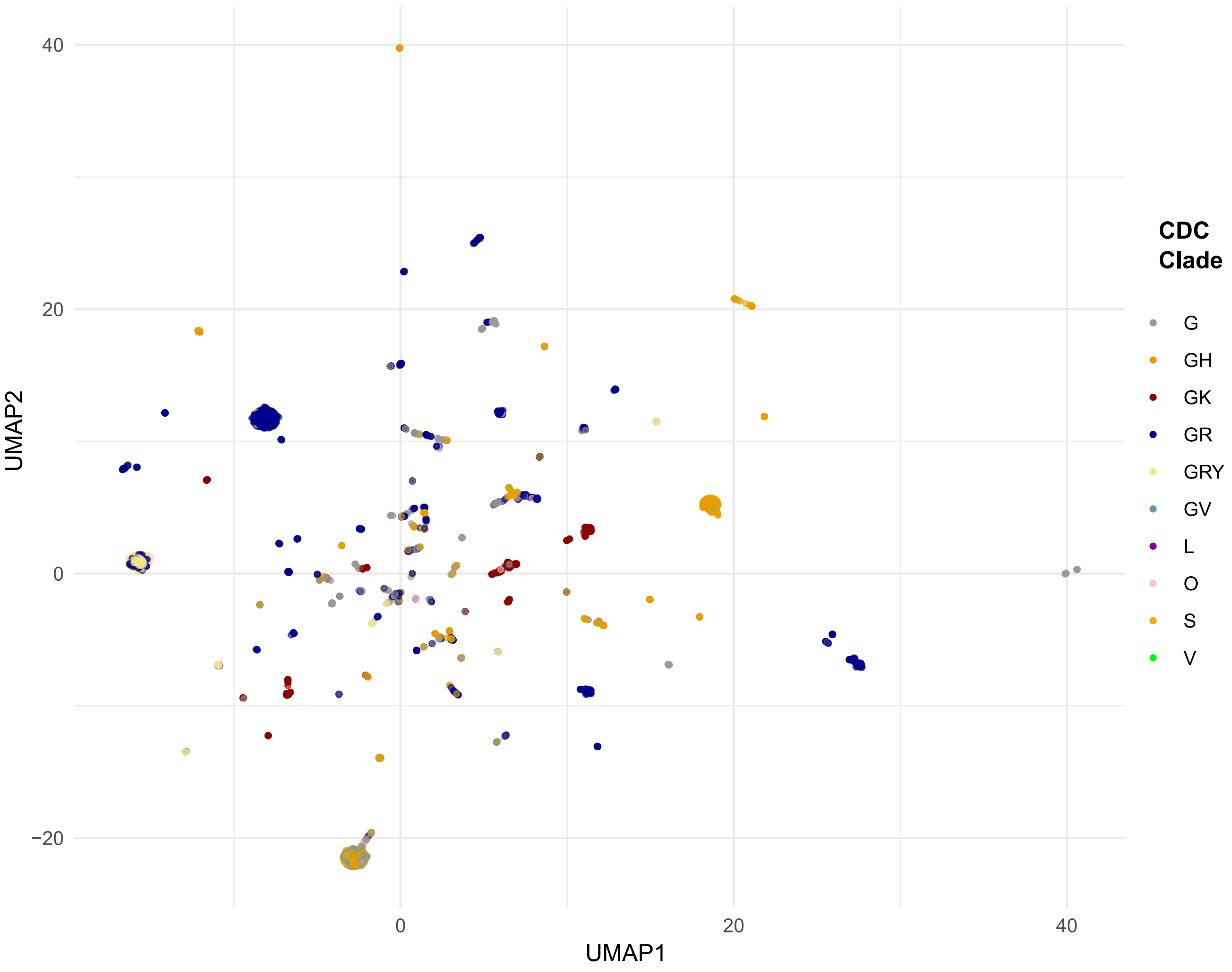

Supplementary Figure S3

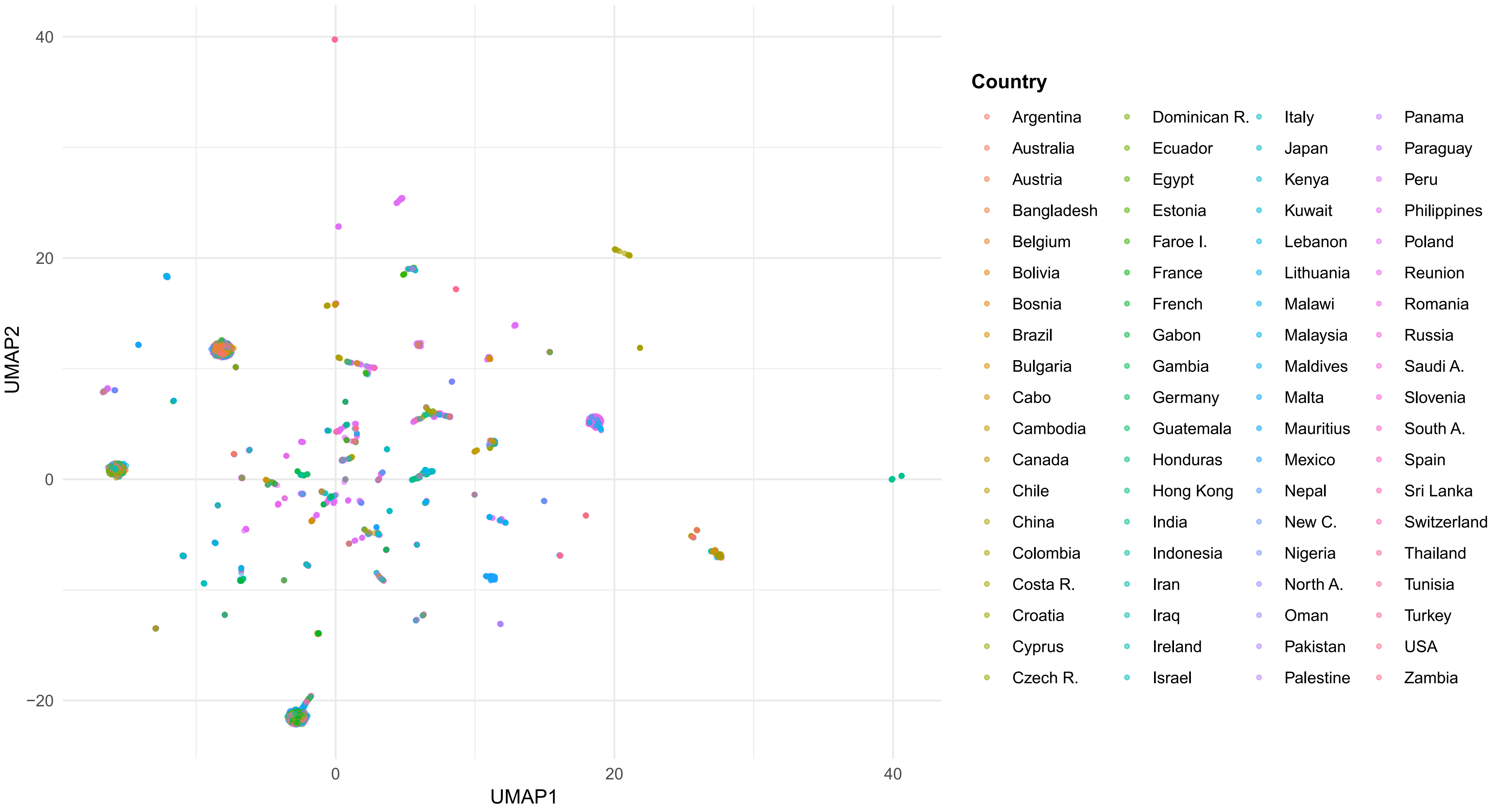

Supplementary Figure S4

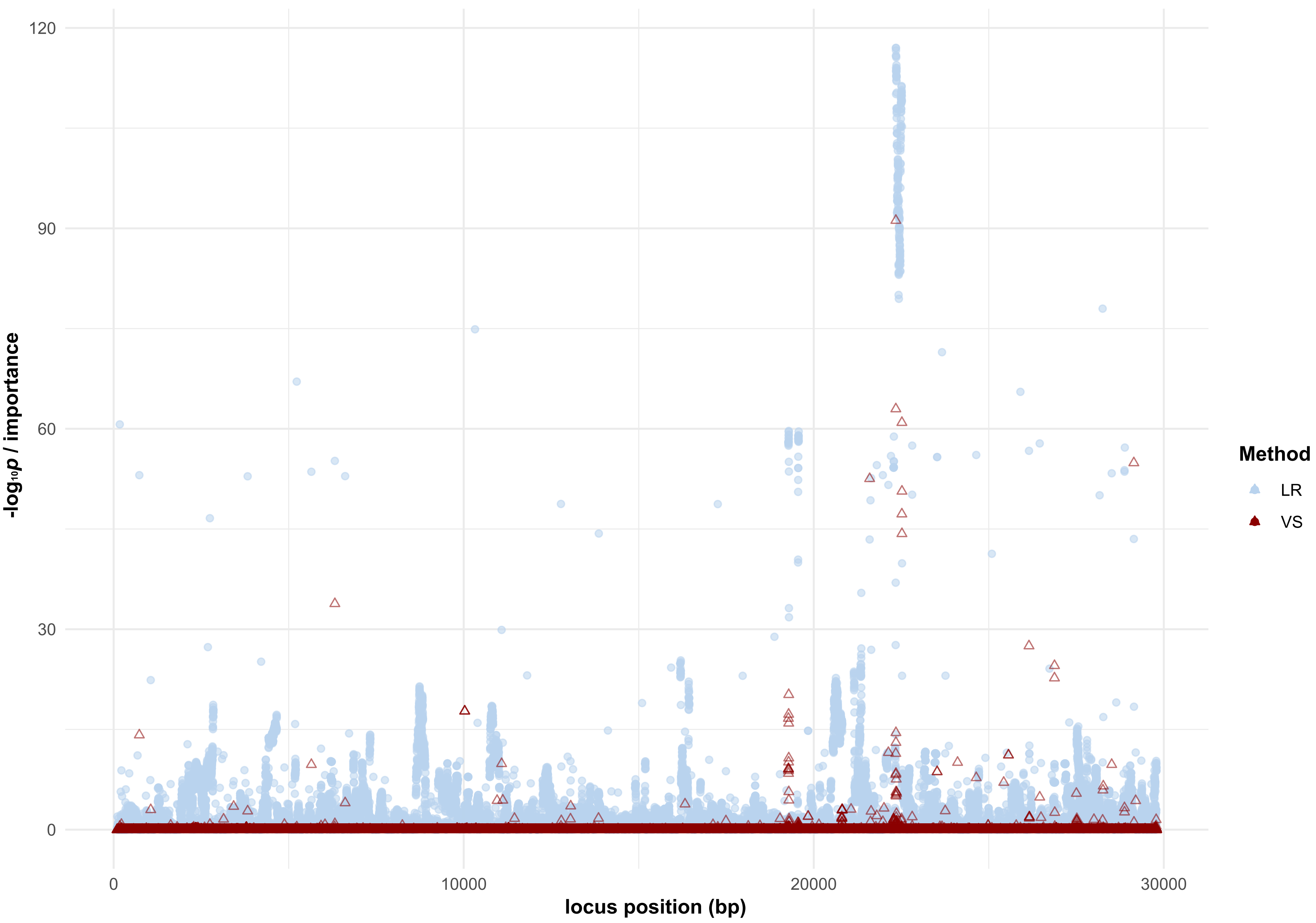

Supplementary Figure S5

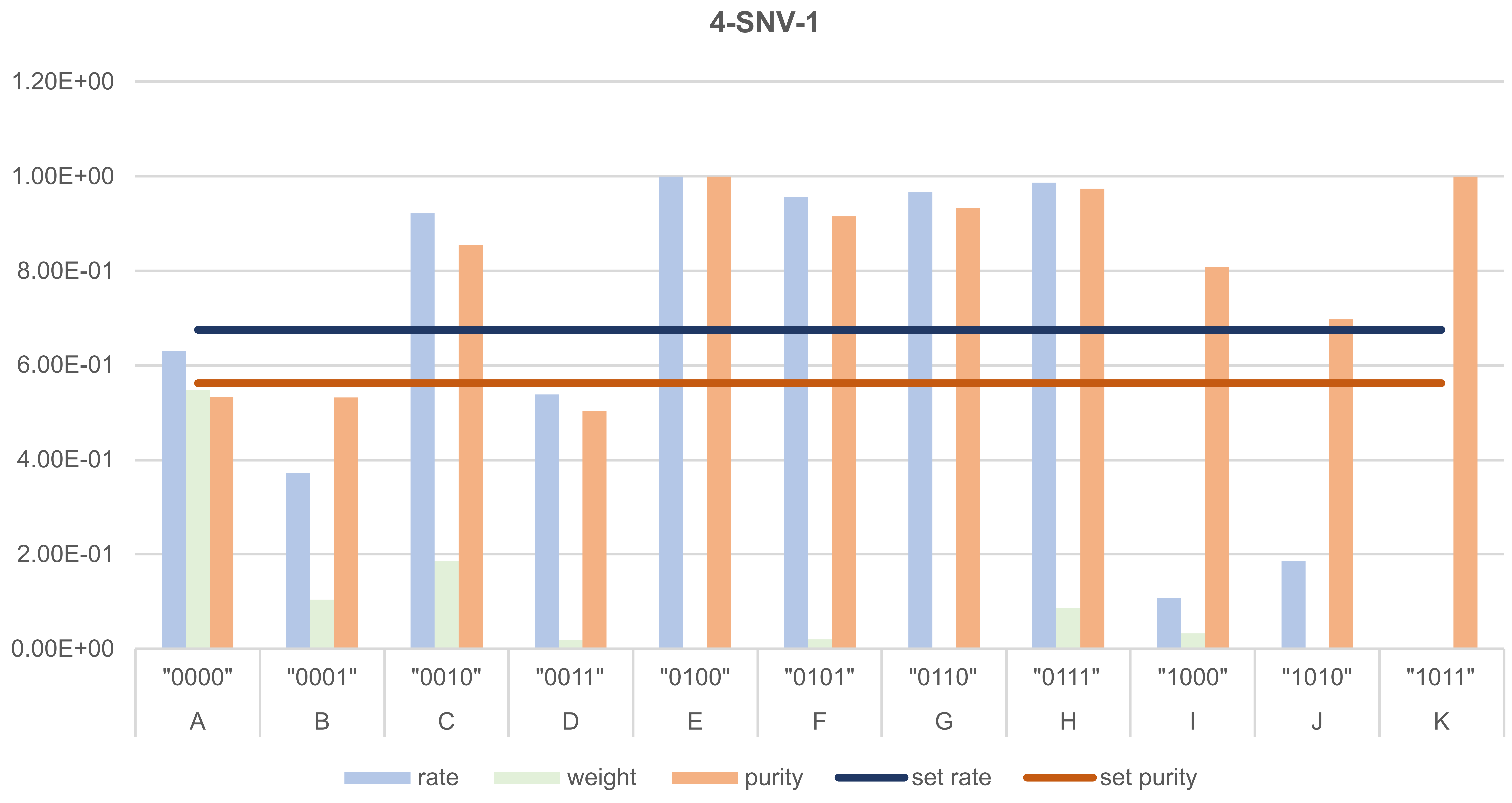

Supplementary Figure S6

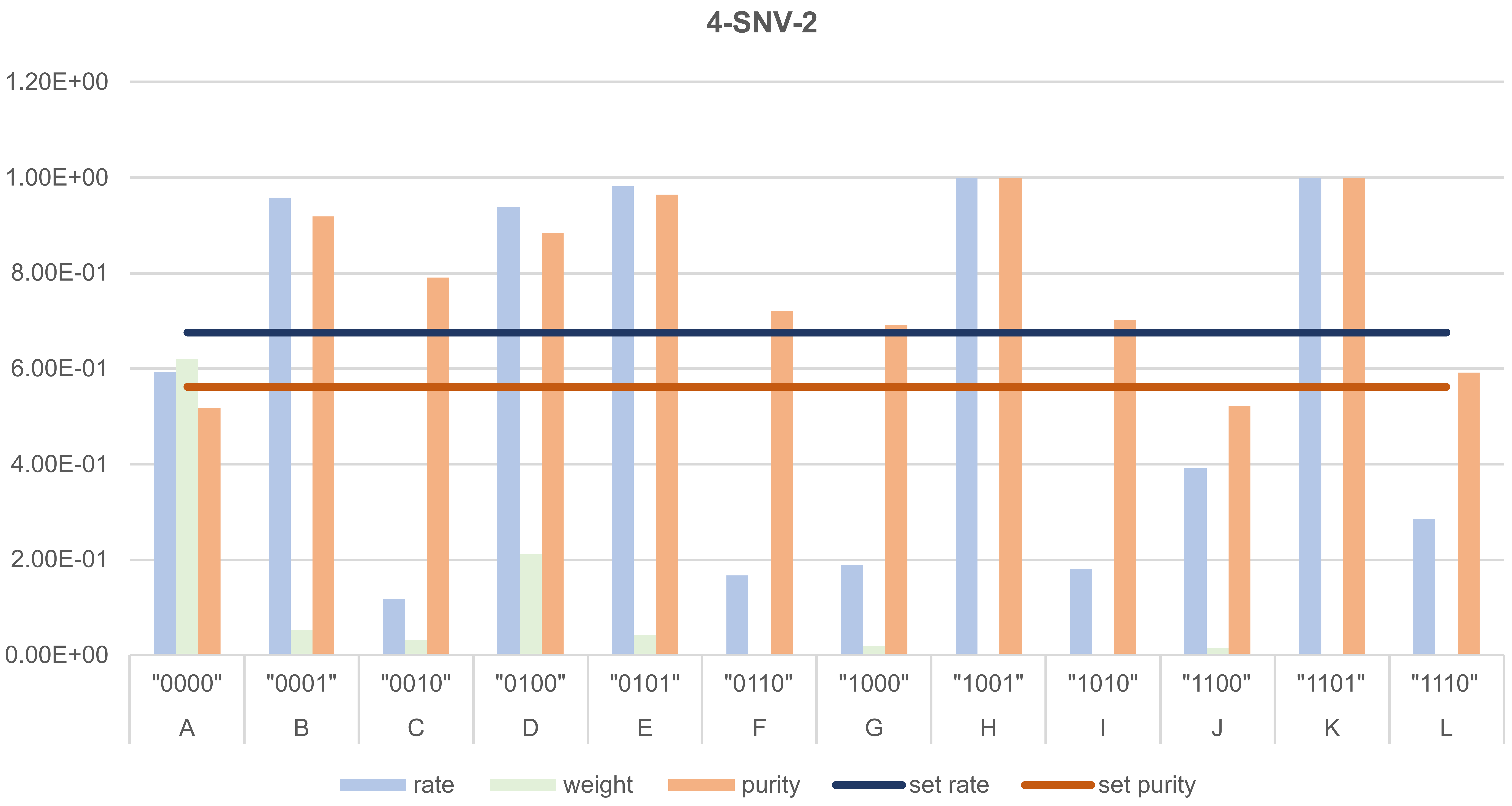

Supplementary Figure S7

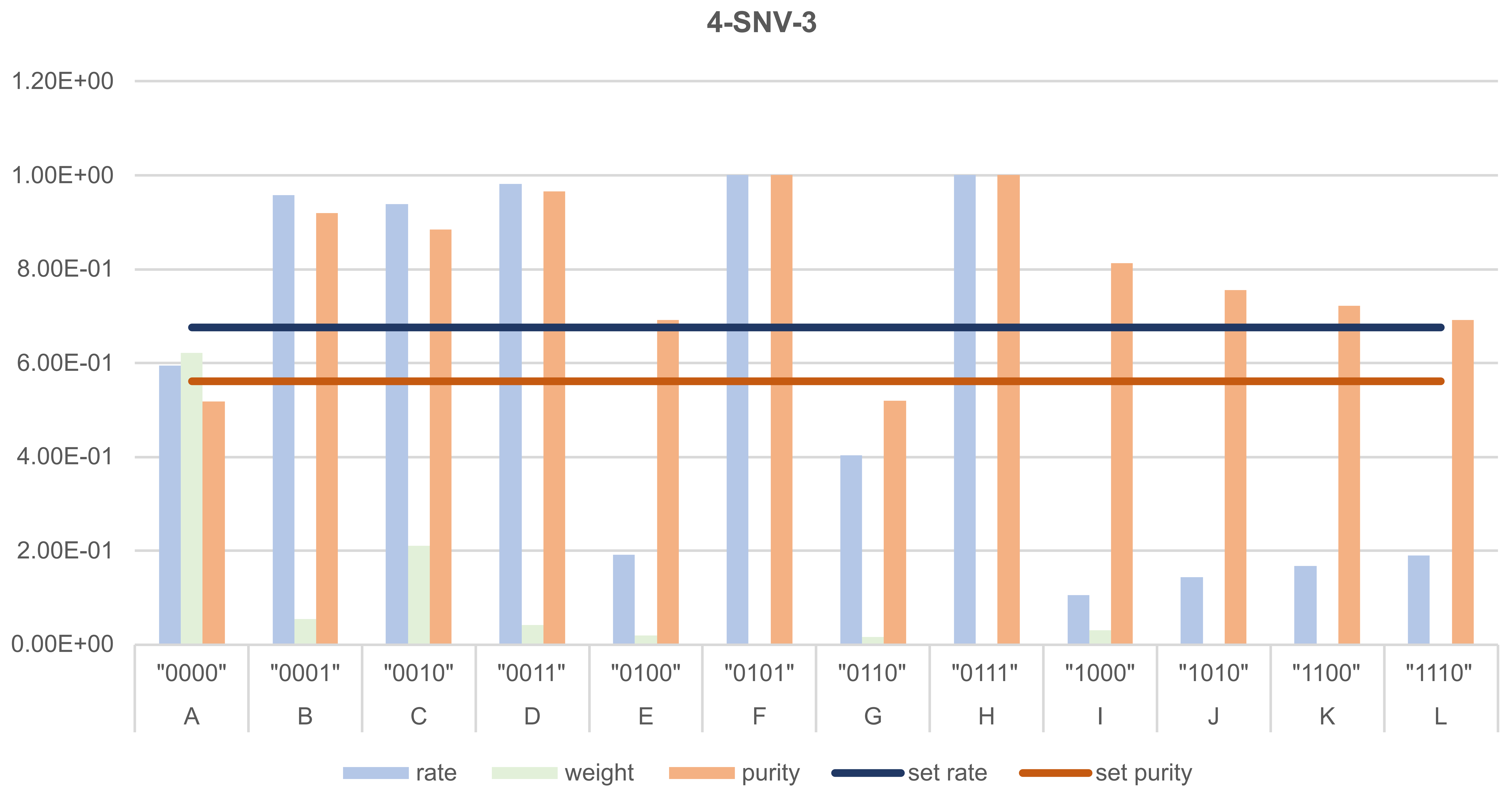

Supplement: Supplementary data 2 [file mmc2.pdf]
